# Diurnal Temperature Variations Affect Development of a Herbivorous Arthropod Pest and its Predators

**DOI:** 10.1371/journal.pone.0124898

**Published:** 2015-04-15

**Authors:** Dominiek Vangansbeke, Joachim Audenaert, Duc Tung Nguyen, Ruth Verhoeven, Bruno Gobin, Luc Tirry, Patrick De Clercq

**Affiliations:** 1 Laboratory of Agrozoology, Department of Crop Protection, Ghent University, Coupure Links 653, B-9000, Ghent, Belgium; 2 PCS-Ornamental Plant Research, Schaessestraat 18, B-9070, Destelbergen, Belgium; 3 Entomology Department, Vietnam National University of Agriculture, Trau Quy, Gia Lam, Hanoi, Vietnam; Federal University of Viçosa, BRAZIL

## Abstract

The impact of daily temperature variations on arthropod life history remains woefully understudied compared to the large body of research that has been carried out on the effects of constant temperatures. However, diurnal varying temperature regimes more commonly represent the environment in which most organisms thrive. Such varying temperature regimes have been demonstrated to substantially affect development and reproduction of ectothermic organisms, generally in accordance with Jensen’s inequality. In the present study we evaluated the impact of temperature alternations at 4 amplitudes (DTR0, +5, +10 and +15°C) on the developmental rate of the predatory mites *Phytoseiulus persimilis* Athias-Henriot and *Neoseiulus californicus* McGregor (Acari: Phytoseiidae) and their natural prey, the two-spotted spider mite *Tetranychus urticae* Koch (Acari: Tetranychidae). We have modelled their developmental rates as a function of temperature using both linear and nonlinear models. Diurnally alternating temperatures resulted in a faster development in the lower temperature range as compared to their corresponding mean constant temperatures, whereas the opposite was observed in the higher temperature range. Our results indicate that Jensen’s inequality does not suffice to fully explain the differences in developmental rates at constant and alternating temperatures, suggesting additional physiological responses play a role. It is concluded that diurnal temperature range should not be ignored and should be incorporated in predictive models on the phenology of arthropod pests and their natural enemies and their performance in biological control programmes.

## Introduction

Temperature has been recognized to be a key abiotic factor driving population dynamics of arthropods, which has resulted in a plethora of studies on the relationship between arthropod developmental biology and temperature [1–4]. To predict developmental rates of poikilothermic arthropods, both linear and nonlinear models have been developed [5, 6]. Linear models allow the estimation of the lower developmental threshold (i.e. the temperature at which the development rate approaches zero) and the thermal constant for development (expressed in degree-days) [3,7], but fail to predict developmental rates at low and high extreme temperatures [5,8]. Nonlinear models more accurately describe the usually curvilinear relationship between arthropod developmental rate and temperature over the whole temperature range [9–11]. Hitherto, these models were mainly based on data from constant temperatures, which is surprising as in most environments varying temperature regimes are the rule, rather than the exception [12–15]. Diurnal temperature ranges, (hereafter referred to as DTR) have been shown to severely impact developmental rates of poikilothermic arthropods [12,16–19]. Therefore, models incorporating the effects of DTR should increase accuracy of predictions and fine-tune existing models.

Usually, at varying temperature regimes, poikilotherm developmental rate tends to be higher at low temperatures and lower in the higher temperature range, as compared to the corresponding mean constant temperature [2,14]. At intermediate temperatures, little to no difference in developmental rates has been observed [2,20,21]. This effect has been attributed to the typically nonlinear relationship between poikilothermic developmental rates and temperature [10,22], and has been referred to as the rate summation effect or Kaufmann effect [14]. Generally, this phenomenon is a consequence of Jensen’s inequality [23], which states that the average value of a nonlinear function (E[f(*x*)]) of two values of *x* does not necessarily equals the value of the nonlinear function evaluated at the average variable (f(E[*x*]) [24] (see S1 Appendix). This mathematical property may, at least partly, explain the variation in arthropod developmental rates between constant and varying temperature regimes [17,24,25]. Other possible explanations for the observed differences in developmental rates between constant and varying temperature regimes refer to (yet unknown) physiological responses that act in addition to the rate summation effect [2,14,17], or have been attributed to the presence or lack of a diurnal rhythm, as it would occur in the organism’s natural environment [26].

In pest management strategies, knowledge about the basic thermal biology of both pests and natural enemies is crucial to predict and manage pest outbreaks [27–29]. Temperature-driven models are an essential tool for predicting and managing agricultural and horticultural pests [30–31]. Evidently, as temperature regimes affect developmental rates and other life history parameters, DTRs should be included in such models [32,33].

In this study, we focused on the predatory mites *Phytoseiulus persimilis* Athias-Henriot and *Neoseiulus californicus* McGregor (Acari: Phytoseiidae), two natural enemies of the two-spotted spider mite, *Tetranychus urticae* Koch (Acari: Tetranychidae) [34]. The two-spotted spider mite is an extremely polyphagous agricultural pest with an unmatched level of pesticide resistance [35]. In protected crops, introduction of commercial strains of these phytoseiid predators of *T*. *urticae* has shown to be a successful alternative for chemical control [36–38]. Recently, the influence of temperature variations on both pest and predators has been investigated [39,40], revealing a substantial impact on their development, fecundity and population growth. Here, we explored the developmental rates of the mite species under a wider range of temperatures at different DTRs.

Our study investigated the relationship between developmental rate of *P*. *persimilis*, *N*. *californicus* and *T*. *urticae*, and temperature under both constant and alternating temperature regimes at four amplitudes (i.e., DTR of 0, +5, +10 and +15, resulting in a difference of 0, 5, 10 and 15°C between day and night temperatures). We evaluated linear and nonlinear models to predict developmental rates and assessed whether we could use data derived from constant temperatures to predict the effects of alternating temperatures, thereby assessing whether Jensen’s inequality is the main factor explaining the observed differences. Finally, we explore the impact of the mites' responses to these temperature variations on their performance in biological control programmes.

## Materials and Methods

### Mite rearing

Two-spotted spider mites were originally collected from *Ricinus communis* L. plants grown at the Faculty of Bioscience Engineering of Ghent University, Ghent, Belgium. A laboratory colony was maintained on kidney bean plants (*Phaseolus vulgaris* L.) for more than 2 years before the onset of the experiments. Colonies of both phytoseiid species were started with individuals supplied by Biobest N.V. (Westerlo, Belgium) and maintained on reversed kidney bean leaves placed on cotton soaked in water in a petri dish (ø 14 cm) [39]. The edges of the leaves were covered with an additional layer of water-soaked cotton to provide free water and prevent the mites from escaping. Bean leaves were infested with an abundance of mixed stages of *T*. *urticae* as a food source for the predators. All mite colonies were maintained in a climatic cabinet (Sanyo Electric Co., Ltd., Japan) at 25 ± 1°C, 65 ± 5% RH and a 16:8 h (L:D) photoperiod.

### Experimental set-up

The development of *T*. *urticae* and its predators *P*. *persimilis* and *N*. *californicus*, was studied at a 16:8 h (L:D) photoperiod and at different constant and alternating temperature regimes between 12.5 and 40°C with 4 different amplitudes (constant: 0°C and alternating: 5, 10 and 15°C) (S2 Appendix). For the temperature regimes 15°C/15°C, 20°C/5°C and 20°C/20°C, data on developmental rates of both phytoseiids were taken from a previous study [39].

Leaf arenas were infested with *T*. *urticae* 5 days before the introduction of a predatory mite egg by transferring 3 gravid female spider mites to the arena. Hence, an excess amount of both eggs and motile stages of *T*. *urticae* was supplied as a food source for the phytoseiid immatures.For *P*. *persimilis* and *N*. *californicus*, 40 eggs of each species (<6h) were collected from the stock colony and were transferred individually to square bean leaf arenas (25 x 25mm) using a fine needle. The leaf arenas were placed upside down on a water-soaked polyurethane sponge (10 x 50 x 50 mm) in polystyrene insect breeding dishes (ø 100 mm, H 40 mm) (SPL Life Sciences, Korea). Ventilation was provided with a mesh covered hole (ø 40 mm) in the lid. To prevent the mites from escaping and to provide free water, moist tissue paper was used to cover the edges of the bean leaf arenas.

For the experiments with *T*. *urticae*, 3 gravid female spider mites from the stock colony were introduced to each leaf arena as described above 4h prior to the onset of the test. Thereafter, the females were removed and the amount of spider mites eggs was reduced to a single egg per arena by piercing the excess of eggs randomly.

The development of the three mite species was monitored twice a day (at 8 am and 6 pm) when the average daily temperature was equal or higher than 25°C. When the average temperature was lower than 25°C, development was checked daily. The developmental progress was tracked by the presence of exuviae on the leaf disc.

When the developmental period of both phytoseiids and *T*. *urticae* exceeded 10 days, mites were transferred to fresh leaf arenas.

Relative humidity is an additional factor determining the developmental success of *P*. *persimilis*, *N*. *californicus* and *T*. *urticae* [41,42]. Inside the insect breeding dishes, relative humidity was measured using HOBO H8 RH/Temp Loggers (Onset Computer, Bourne, MA, USA) and always exceeded 90%. Therefore, relative humidity during the experiments was assumed not to be a limiting factor for development of the mites.

### Statistical analysis

Data were analyzed using SPSS Statistics (Version 20, IBM). Mean female developmental times were compared using non-parametric Kruskal-Wallis ANOVAs as data were found not to be normally distributed. Means were separated using Mann-Whitney tests. The level of significance was set at 0.05.

### Modelling

For further analysis, we only used female developmental rates (D_r_, in day^-1^) and were derived by calculating the reciprocal of the developmental times (D) obtained from the experiments. Developmental rates were subjected to both linear and nonlinear regression. To describe the nonlinear relationship between developmental rate and temperature, a variety of functions have been constructed with different levels of complexity, numbers of parameters, different assumptions about high and low temperature limits and inclusion of biologically relevant parameters, such as optimal temperature (T_opt_) and upper and lower developmental threshold (T_L_ and T_0_, respectively) [1,6,43]. We selected two nonlinear equations with a low level of complexity, which predict biologically relevant parameters and have the ability to intersect with the x-axis, thereby allowing an estimation of the lower developmental threshold, namely the Brière-2 and Lactin-2 model [11,43] using SigmaPlot version 12 (SYSTAT Software Inc.).

#### Linear regression

Data that deviated from the straight line were omitted for calculation of the linear regression model [5,44].

$$D_{r}\;=\;a\;+\;b\;*\;T$$ 1

with

- D_r_ = developmental rate (day^-1^)
- T = temperature (°C)
- a = developmental rate when T is 0°C
- b = slope of the regression line

The lower developmental threshold (T_0_) was estimated from the linear model as the intercept of the developmental rate-temperature curve with the temperature axis. The standard error (SE) of T_0_ can be calculated using the following formula [5]:

$$SE_{T_{0}}\;=\frac{r}{b}\sqrt{\frac{s²}{N*r²}+{\left(\frac{SE_{b}}{b}\right)}^{2}}$$ 2

where s² is the residual mean square of D_r_, r is the sample mean and N is the sample size.

The thermal constant (K) indicates the amount of thermal units (in degree-days) that are needed to complete development and can be derived from the linear model as the reciprocal of the slope b (K = 1/b). The SE of K can be estimated as follows [5,6]:

$$SE_{K}=\frac{SE_{b}}{b^{2}}$$ 3

#### Nonlinear regression

Brière-2

$$D_{r}=a\;*\;T\;*\;\left(T-T_{0}\right)\;*\;{\left(T_{L}-T\right)}^{\frac{1}{d}}$$ 4

with

- D_r_ = developmental rate (day^-1^)
- T = temperature (°C)
- a, d = empirical constants
- T_0_ = low temperature developmental threshold (°C)
- T_L_ = lethal temperature threshold (°C)

#### Nonlinear regression

Lactin-2

$${\text{D}}_{r}=e^{\left(\rho *T\right)}-e^{\left(\rho T_{L}-\left(\frac{T_{L}-T}{\Delta T}\right)\right)}+\lambda$$ 5

with

- D_r_ = developmental rate (day^-1^)
- T = temperature (°C)
- ρ = constant defining developmental rate at optimal temperature
- λ = constant forcing the curve to intercept with the x-axis, thereby allowing an estimation of the lower developmental threshold T_0_
- ΔT = temperature range between T_opt_ and T_L_
- T_L_ = lethal maximum temperature

The optimal temperature (T_opt_) is the temperature at which the developmental rate reaches its highest value and was calculated from the first derivative of the above-mentioned nonlinear functions (as the value of T when d(D_r_)/d(T) = 0).

### Model evaluation

The quality of the tested models was evaluated by means of the adjusted R² (R²_adj_) and Akaike’s information criterion (AIC) [45] in addition to R² (coefficient of determination) and RSS (residual sum of squares) by using the following formulae:

$$R_{adj}^{2}=1-\left(\frac{n-1}{n-p}\right)\;*\;\left(1-R^{2}\right)$$ 6

and

$$AIC\;=\;n\;*\;\text{ln}\;\left(\frac{RSS}{n}\right)+2\;*\;p$$ 7

where n is the number of observations, p equals the number of model parameters and RSS is the residual sum of squares. Higher R²_adj_ and lower AIC values, indicate better fits of the model with observed developmental rates. When accepting that the observed differences between varying and constant temperatures are exclusively due to the rate summation effect or Jensen’s inequality based on the curvilinear relationship between temperature and developmental rate, it should be possible to calculate the amount of development by accumulating the proportion of development per time-unit using the following formula [14,17]:

$$D_{r,exp}=\sum_{t=a}^{b}D_{r,obs}\;\left[T\left(t\right)\right]dt$$ 8

where developmental rate D_r, exp_ (developmental rate as expected by the rate summation) is a function of temperature (T), which in turn is a function of time (t), r is the corresponding developmental rate (r = 1/D), and a and b are the start and end, respectively, of the developmental period under a given temperature regime (here a = 0h and b = 24h). D_r,obs_ are the observed developmental rates as calculated by the reciprocal of developmental time D. For example, the expected developmental rate at 25°C/15°C (i.e. DTR+10) can be calculated as follows:

Dr,exp(25°C/15°C)=16/24*Dr,obs,25°C+8/24*Dr,obs,15°C

where D_r,exp_ (25°C/15°C) is the expected developmental rate when accepting the rate summation effect, and D_r, obs, 25°C_ and D_r, obs, 15°C_ are the observed developmental rates at a constant 25°C and 15°C, respectively, and a 16:8 h (L:D) photoperiod.

Next, we compared the obtained expected developmental rates with the observed developmental rates at a given temperature regime as follows [17]:

$$d=\left(\frac{D_{r,exp}}{D_{r,obs}}-1\right)\;*\;100$$ 9

where d equals the percentage deviation. A negative value of d indicates that the rate summation effect underestimates the actual developmental rate, whereas a positive value indicates that the rate summation effect predicts higher developmental rates than what is observed. The higher the deviation, the more we can assume that the observed difference is not solely due to the rate summation effect, but that an additional physiological response is present and that the developmental rate at a specific temperature is not independent of the present temperature regime [14,17].

### Potential impact on biological control

To assess the potential impact of the investigated temperature variations on the dynamics between *P*. *persimilis*, *N*. *californicus* and *T*. *urticae*, we calculated the ratio of the developmental rates at DTR+5, +10 and +15 and that at constant temperature (ΔD_r_ = [D_r_ (alternating T)/ D_r_ (constant T)]) as predicted by the Brière-2 nonlinear model. We selected the latter nonlinear model as generally lower R²_adj_ and AIC values were obtained than for the Lactin-2 model (S3 Appendix). A species will be positively or negatively affected by the alternating temperature regime if ΔD_r_ is higher or lower than 100%, respectively. For fast developing species, changes in developmental period have a greater effect on population growth than a similar proportionate change in reproduction [46]. Therefore, differences in developmental rate will benefit one species over another in terms of population growth and can thus affect the outcome of a biological control programme. Here, we compared the developmental rate of the predatory mites with their prey, *T*. *urticae*, at alternating versus constant temperature regimes.

For each amplitude, we plotted the value of ΔD_r_ (using the Brière-2 nonlinear model) in a temperature range between 10 and 36°C using an interval of 1°C.

## Results

Total developmental periods (egg-adult) of *P*. *persimilis*, *N*. *californicus* and their prey *T*. *urticae* are shown in S1 Appendix. All data are available in S1 Data. Temperature affected the developmental times of all mite species (Kruskal-Wallis: *P*. *persimilis*: χ² = 737.956; df = 29; p<0.001, *N*. *californicus*: χ² = 728.697; df = 33; p<0.001; *T*. *urticae*: χ² = 827.341; df = 33; p<0.001). Immature *P*. *persimilis* were not able to reach adulthood at constant temperatures at or above 35°C, whereas *N*. *californicus* and *T*. *urticae* succeeded in completing development at a daytime temperature of 37.5°C as long as a colder nighttime temperature was maintained.

### Linear regression

**Fig 1:**
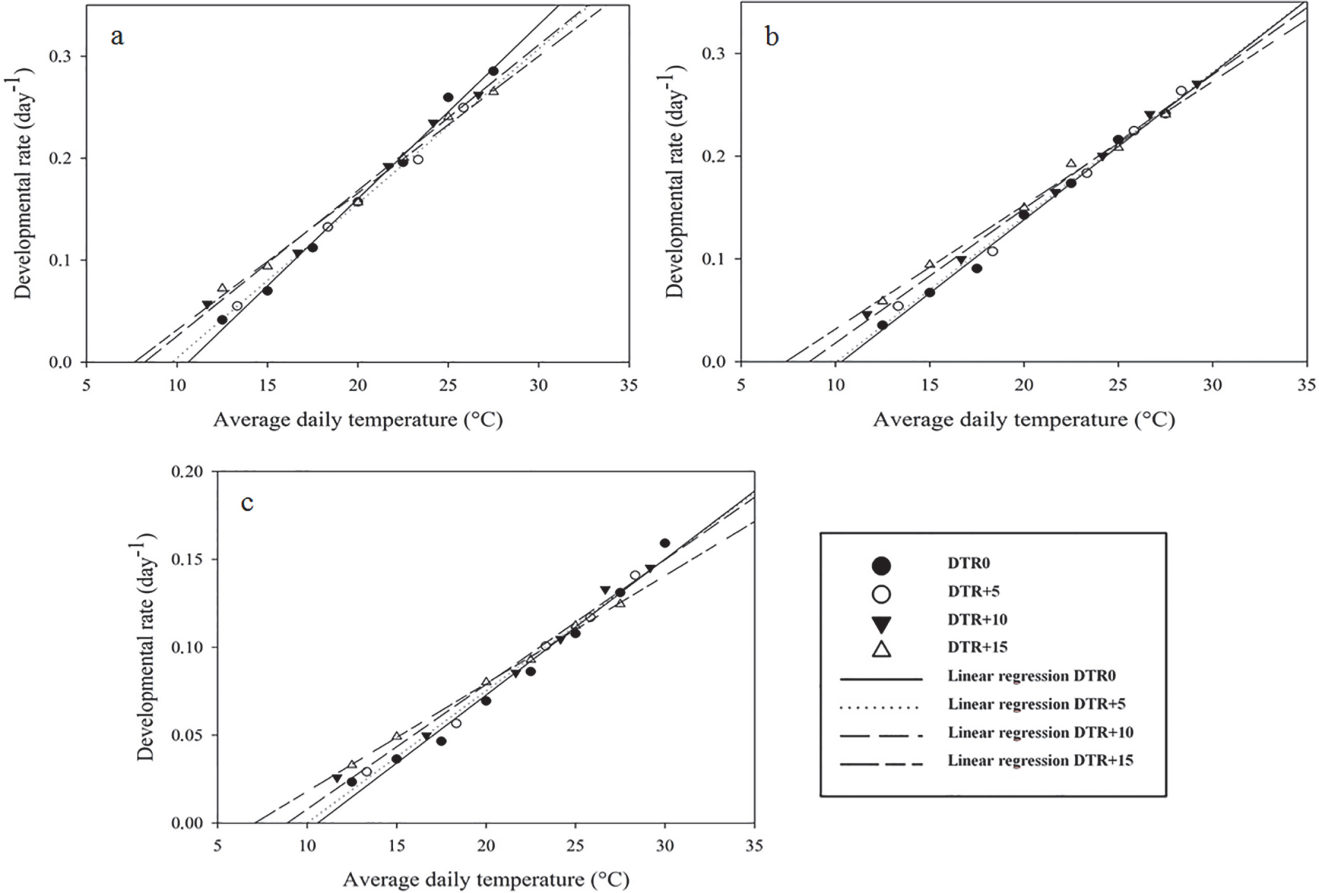
Linear regression of egg-adult developmental rate versus temperature for *Phytoseiulus persimilis* (a), *Neoseiulus californicus* (b) and *Tetranychus urticae* (c) exposed to different constant and alternating temperature regimes

**Fig 2:**
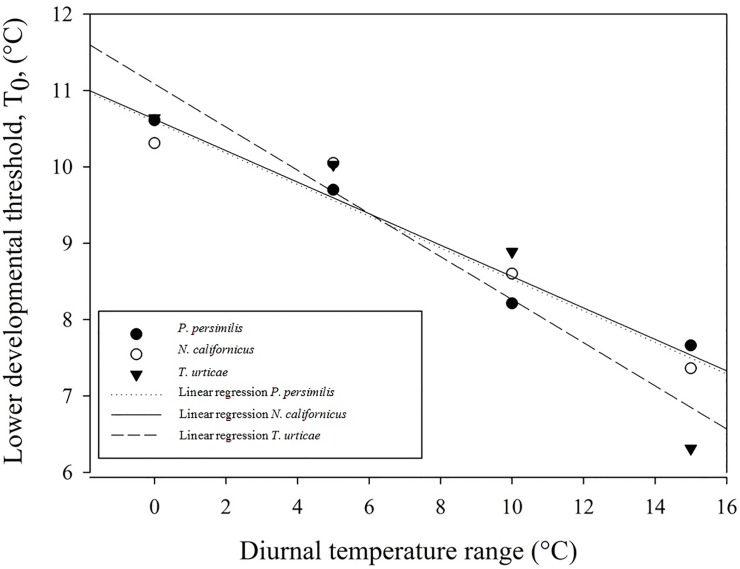
Linear relationship between lower developmental threshold (T_0_) and diurnal temperature range for *Phytoseiulus persimilis*, *Neoseiulus californicus* and *Tetranychus urticae* exposed to different constant and alternating temperature regimes.

When developmental rates at the highest temperatures were omitted from the regression analysis, the linear model showed a good fit to the data (Fig 1), as demonstrated by high values of R² and R²_adj_ (all >0.98) and low values of RSS (Table 1). Diurnal temperature range had an effect on the lower developmental thresholds for egg-adult development of all mite species, with lower T_0-_values with increasing DTR (Fig 2). T_0_-values were about 3°C lower at a DTR+15 temperature regime as compared to the constant temperature regime for all species. When lower developmental thresholds decreased, the thermal constants increased (Table 1).

**Table 1:** Fitted parameters of linear regression (D_r_ = a+b*T) of developmental rates, developmental threshold (T_0_) and thermal constant (K) for total immature development of *Phytoseiulus persimilis*, *Neoseiulus californicus* and *Tetranychus urticae* at 4 thermoperiods (DTR).

Species	DTR (°C)	a	b	R²	R²_adj_	RSS	T_0_ (°C)	K (DD)
*P*. *persimilis*	0	-1.814 ± 0.0140	0.0171 ± 0.0007	0.9921	0.9906	0.0004	10.61 ± 0.43	58.48 ± 2.39
	5	-0.1475 ± 0.0147	0.0152 ± 0.0007	0.9957	0.9935	0.00009	9.70 ± 0.53	65.79 ± 3.03
	10	-0.1174 ± 0.0172	0.0143 ± 0.0008	0.9901	0.9869	0.0003	8.21 ± 0.74	69.93 ± 3.91
	15	-0.1026 ± 0.0121	0.0134 ± 0.0006	0.9928	0.991	0.0002	7.66 ± 0.62	74.63 ± 3.34
*N*. *californicus*	0	-0.1474 ± 0.0107	0.0143 ± 0.0005	0.9934	0.9921	0.0002	10.31 ± 0.38	69.93 ± 2.44
	5	-0.1427 ± 0.0154	0.0142 ± 0.0007	0.9931	0.9907	0.0002	10.05 ± 0.63	70.42 ± 3.47
	10	-0.1126 ± 0.0091	0.0131 ± 0.0004	0.9962	0.9952	0.0001	8.60 ± 0.44	76.34 ± 3.42
	15	-0.089 ± 0.0098	0.0121 ± 0.0005	0.9941	0.9926	0.0001	7.36 ± 0.57	82.64 ± 3.42
*T*. *urticae*	0	-0.0819 ± 0.0091	0.0077 ± 0.0004	0.9831	0.9803	0.0003	10.64 ± 0.64	129.87 ± 6.75
	5	-0.0752 ± 0.0089	0.0075 ± 0.0004	0.9918	0.9891	0.00007	10.03 ± 0.69	133.33 ± 7.11
	10	-0.0631 ± 0.0062	0.0071 ± 0.0004	0.9856	0.982	0.0002	8.89 ± 0.80	140.85 ± 7.93
	15	-0.0366 ± 0.0061	0.0058 ± 0.0003	0.989	0.9868	0.00009	6.31 ± 0.84	172.41 ± 8.92

### Nonlinear regression

**Fig 3:**
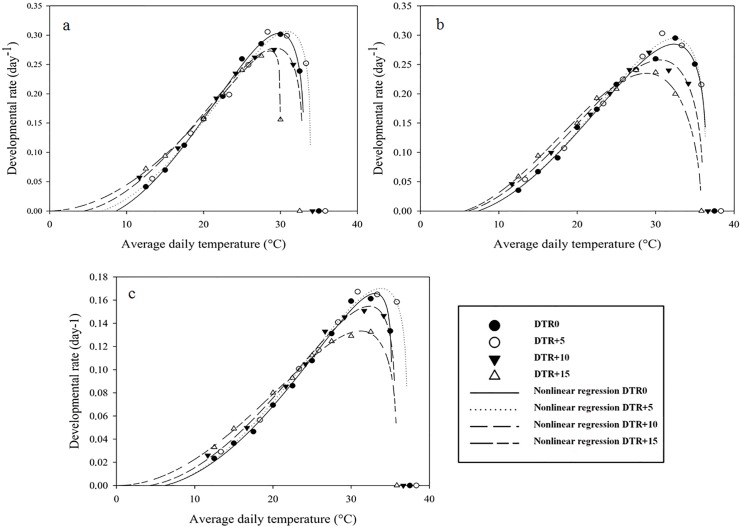
Nonlinear regression (Brière-2) of egg-adult developmental rate versus temperature for *Phytoseiulus persimilis* (a), *Neoseiulus californicus* (b) and *Tetranychus urticae* (c) exposed to different constant and alternating temperature regimes.

**Fig 4:**
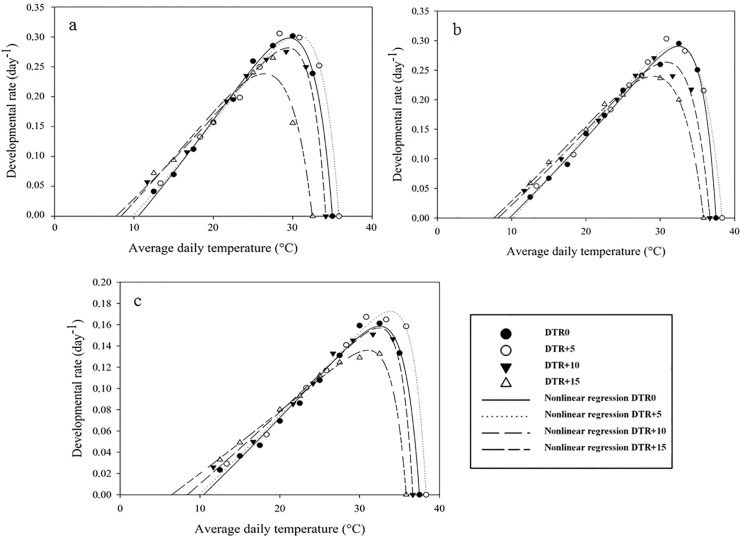
Nonlinear regression (Lactin-2) of egg-adult developmental rate versus temperature for *Phytoseiulus persimilis* (a), *Neoseiulus californicus* (b) and *Tetranychus urticae* (c) exposed to different constant and alternating temperature regimes.

Nonlinear models fitted the data well (Figs 3 and 4), as reflected by the high R² and R²_adj_ and low RSS and AIC values (S3 Appendix). A similar trend as for the linear models was observed regarding the effect of DTR on the low temperature developmental threshold, with decreasing T_0_-values as the difference between day and night temperatures increased. In general, lethal temperatures decreased with an increasing DTR. Optimal temperatures, calculated by the first derivative of the model equation, were higher at DTR+5 than at a constant temperature (DTR0). For *P*. *persimilis* and *T*. *urticae*, optimal temperatures at DTR+5 were about 1°C higher than at DTR0, whereas for *N*. *californicus* the relationship between optimal temperatures at constant and alternating temperatures was less clear.

### Contribution of the rate summation effect to observed differences in developmental rates at constant and alternating temperatures

The percent deviation values shown in Table 2, Table **3** and Table 4 indicate that it is not possible to use developmental rates obtained at constant temperatures to accurately predict the rates at alternating temperatures over the whole temperature range. The deviation is more pronounced at lower and higher average temperatures, with values of over 50% in the higher temperature range. At intermediate average temperatures, the percent deviation was overall low (< 10%). Thus, a physiological response that acts in addition to the rate summation effect can be expected in the lower and higher temperature range.

**Table 2:** Percent deviation (d) of expected (according to the rate summation effect) from observed developmental rates at a DIF+5 temperature regime and a 16L:8D h photoperiod for *Phytoseiulus persimilis*, *Neoseiulus californicus* and *Tetranychus urticae*.

Temperature (°C)			d (%)		
Day	Night	Daily average	*P*. *persimilis*	*N*. *californicus*	*T*. *urticae*
15	10	13.3	-14.9	-16.9	-16.6
20	15	18.3	-2.8	10.2	3.2
25	20	23.3	13.8	4.942	-6.3
27.5	22.5	25.8	2.7	-2.5	-0.7
30	25	28.3	-5.1	-3.1	0.8
32.5	27.5	30.8	-14.0	-7.7	-9.6
35	30	33.3	-59.8	-9.7	-14.3
37.5	32.5	35.8	/^a^	-53.3	-66.1

^a^ Immatures were not able to develop to adults.

**Table 3:** Percent deviation (d) of expected (according to the rate summation effect) from observed developmental rates at a DIF+10 temperature regime and a 16L:8D h photoperiod for *Phytoseiulus persimilis*, *Neoseiulus californicus* and *Tetranychus urticae*.

Temperature (°C)			d (%)		
day	night	Average	*P*. *persimilis*	*N*. *californicus*	*T*. *urticae*
15	5	11.7	-18.0	-2.5	-6.3
20	10	16.7	-1.8	-4.2	-7.2
25	15	21.7	2.7	1.5	-1.8
27.5	17.5	24.2	8.4	-4.5	-1.7
30	20	26.7	-12.8	-8.0	-2.8
32.5	22.5	29.2	-17.8	-5.1	-6.3
35	25	31.7	-65.0	0.4	-17.3
37.5	27.5	34.2	/ ^a^	-62.4	-70.1

^a^ Immatures were not able to develop to adults.

**Table 4:** Percent deviation (d) of expected (according to the rate summation effect) from observed developmental rates at a DIF+15 temperature regime and a 16L:8D h photoperiod for *Phytoseiulus persimilis*, *Neoseiulus californicus* and *Tetranychus urticae*.

Temperature (°C)			d (%)		
day	night	Average	*P*. *persimilis*	*N*. *californicus*	*T*. *urticae*
17.5	2.5	12.5	3.7	3.7	-5.7
20	5	15	12.1	1.4	-5.8
25	10	20	10.9	-3.1	-10.2
27.5	12.5	22.5	2.0	-10.1	2.5
30	15	25	-5.6	-5.6	5.4
32.5	17.5	27.5	-25.1	-5.0	-1.2
35	20	30	-66.0	-8.8	-13.2
37.5	22.5	32.5	/ ^a^	-70.9	-78.4

^a^ Immatures were not able to develop to adults.

### Potential impact on biological control

**Fig 5:**
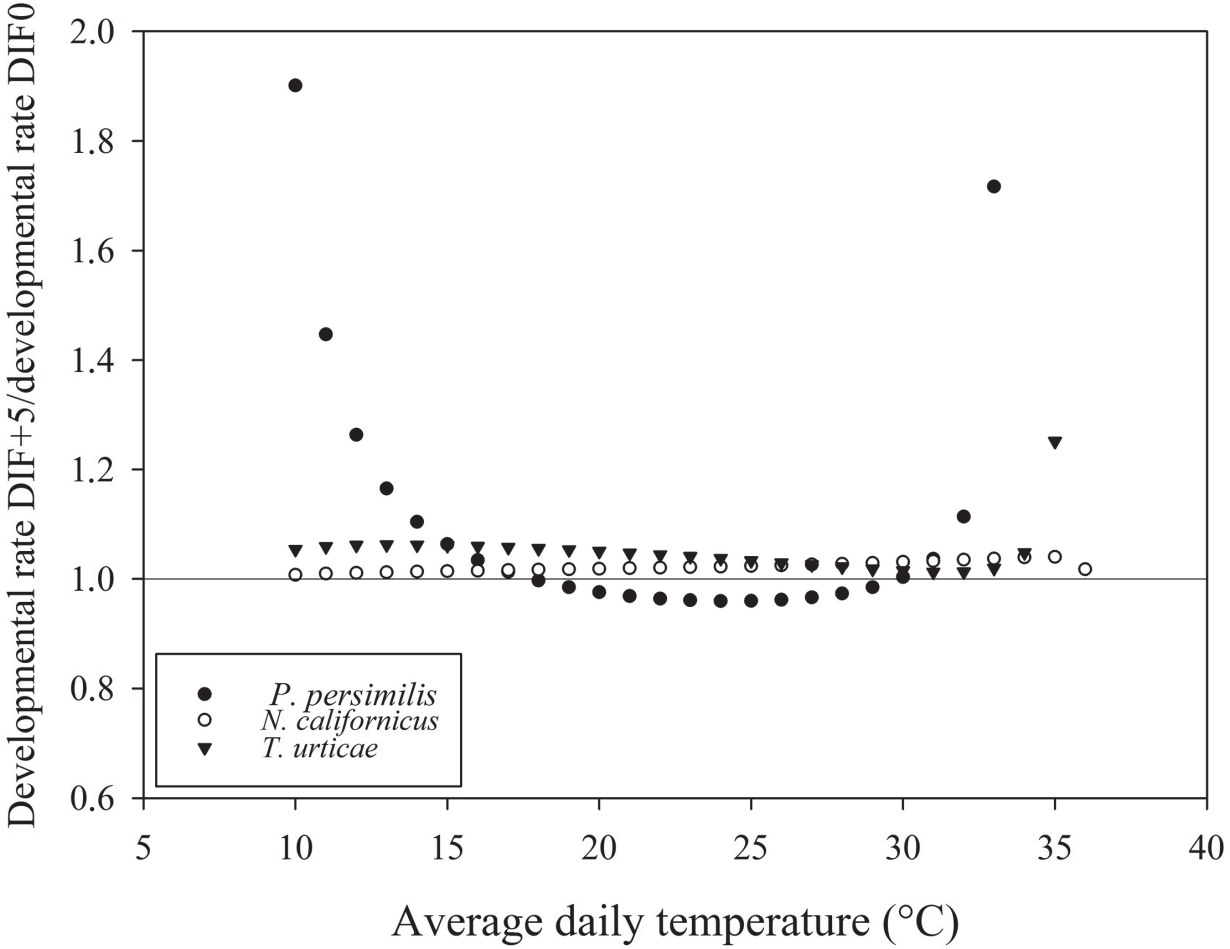
Ratio of developmental rate between DTR+5 and DTR0 for *Phytoseiulus persimilis*, *Neoseiulus californicus* and *Tetranychus urticae*.

**Fig 6:**
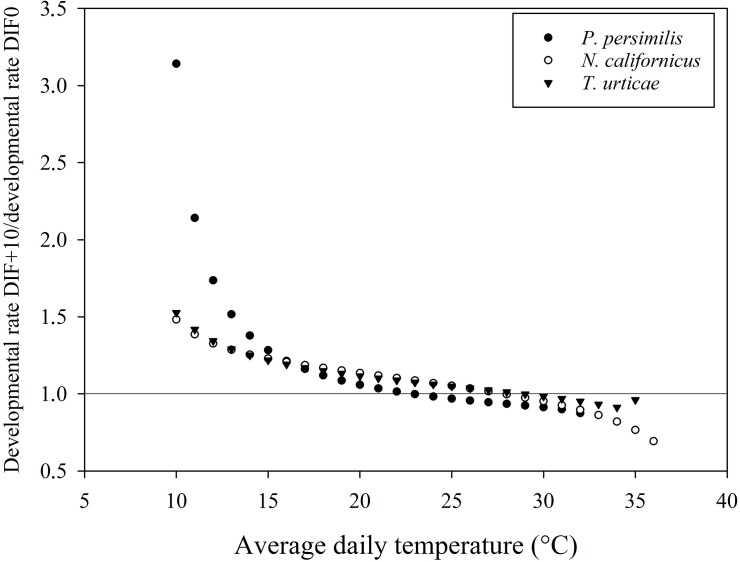
Ratio of developmental rate between DTR+10 and DTR0 for *Phytoseiulus persimilis*, *Neoseiulus californicus* and *Tetranychus urticae*.

**Fig 7:**
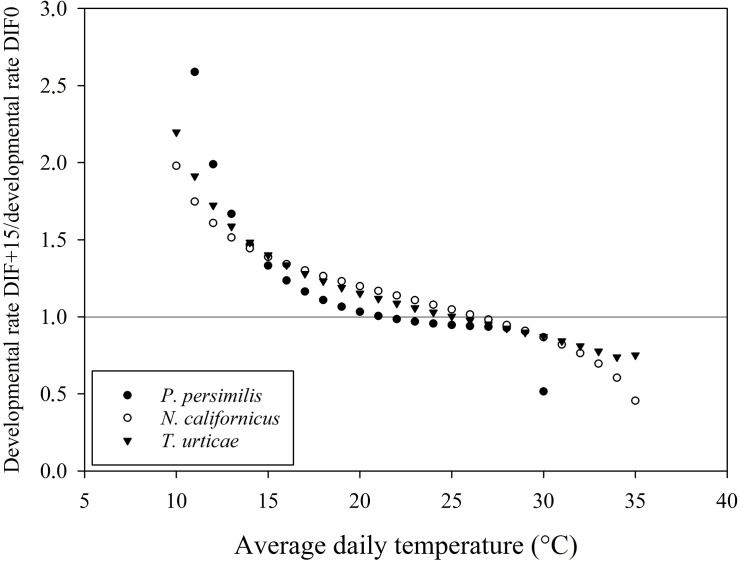
Ratio of developmental rate between DTR+15 and DTR0 for *Phytoseiulus persimilis*, *Neoseiulus californicus* and *Tetranychus urticae*.

As shown in Figs 5, 6 and 7, many points deviated from the line at ratio 1.0 (i.e. the ratio at which a similar developmental rate was predicted for a constant temperature (DTR0) versus a DTR of 5, 10 and 15°C, respectively). Temperature variations resulted in interspecifically different responses in developmental rate. For example, a DTR+5 temperature regime resulted in a faster development of the phytoseiid predator *N*. *californicus* and of its prey, *T*. *urticae*, than at the corresponding mean constant temperature in a range between 20°C and 30°C. For the other predatory mite *P*. *persimilis*, however, development in the temperature range between 20 and 30°C was always faster at the constant temperature regime. When mean temperatures dropped below 15°C, *P*. *persimilis* benefited more from temperature variations than *N*. *californicus* and *T*. *urticae* at each tested DTR.

## Discussion

Temperature alternations had a substantial impact on the egg-adult developmental rates of the phytoseiid predators *P*. *persimilis* and *N*. *californicus* and their prey *T*. *urticae* as compared to the rates at the corresponding mean constant temperatures. In line with earlier studies on thermal responses of arthropods [2,14,15,17,21] developmental rates were higher at varying temperatures in the lower temperature range, whereas lower developmental rates were observed at higher temperatures compared to the corresponding constant temperature regimes. However, not all deviations could be explained by the rate summation effect (see Tables 2, 3 and 4). Intriguingly, the highest developmental rates were observed at a DTR+5 and not at an optimal constant temperature. A direct consequence of the rate summation effect-and therefore also of Jensen’s inequality rule- is that a weighted average (16h light and 8h dark) of developmental rates at constant temperatures used to predict rates at alternating temperatures can never exceed the maximum rate at optimal constant temperature. However, for *P*. *persimilis*, *N*. *californicus* and *T*. *urticae*, alternating temperatures with an amplitude of 5°C (29.2°C/24.2°C, 30.8°C/25.8°C and 32.1/27.1°C, respectively) resulted in a faster development than the highest rate at the optimal constant temperature. This is, at least for the species tested in this study, an indication that rate summation might be insufficient to explain the observed differences between developmental rates obtained at constant and alternating temperature regimes.

The paradoxical idea that a temperature lower than T_opt_ is the temperature at which fitness is maximized was discussed by Martin and Huey [47]. As the asymmetric temperature-rate curve of ectothermic organisms rapidly declines when temperatures exceed the optimal temperature, a slight increase in temperature above T_opt_ has a tremendous detrimental effect on the development rate, whereas a similar slight decrease below T_opt_ has relatively little impact. Therefore, ectotherms might experience an increased fitness at a temperature somewhat lower than T_opt_ instead temperatures higher than T_opt_, which cause a corresponding drop in developmental rate (“suboptimal is optimal”, [47]).

Possible additional physiological mechanisms along with to the rate summation effect have been reported, albeit explained vaguely [14,17,48] Some authors [2, 49] have attributed these responses to a disorganized metabolism, an inadequate supply of nutrients and oxygen, or desiccation; the latter cause does not apply to our study as our experiments were conducted at high humidity (>90% RH). Behrens et al. [26] pointed out that ectothermic organisms have evolved in an environment with fluctuating temperature and are therefore adapted to diurnally changing temperatures. Therefore, the lack of a diurnal rhythm (i.e. diurnal periodicity of activity and rest) per se might affect certain metabolic reactions. Additionally, the energy demand during the day is likely to be higher than during the night, as the optimal temperature for metabolic reactions during the night is lower than that during the light phase which is probably the more active part of the day [2]. For *T*. *urticae*, a higher activity (feeding and oviposition) was observed during the day (light) than during the night (dark) [50]. In the same study, the predatory mite *Amblyseius womersleyi* Schicha (Acari: Phytoseiidae) showed a similar activity pattern, with reduced dispersal and predation rates during the night. Accordingly, we found a lower hourly predation rate of *P*. *persimilis* and *N*. *californicus* on *T*. *urticae* eggs during the night than during the day at a constant temperature of 20°C (Vangansbeke et al., unpublished data). As a result, if the food requirements are satisfied during the day, energy demands (such as respiration) during periods of rest should be lower under alternating temperatures than under corresponding constant temperatures [2].

Other possible mechanisms that might act in addition to the rate summation effect, may be found in the production of cryoprotectants when the organism is exposed to low night temperatures [51] or heat shock proteins when exposed to high daytime temperatures [52,53]. Revealing such mechanisms warrants further analysis at the molecular level. Recently, the differentially expressed genes of diapausing versus non-diapausing *T*. *urticae* females were documented [54]. Similarly, such expression analysis could reveal which genes are differentially expressed when temperatures are allowed to vary instead of being kept constant. Possibly, the expression of certain genes is triggered by a diurnal rhythm [55]. Additionally, we cannot exclude indirect effects of the DTR on the development of *T*. *urticae* via nutritional value of the leaf discs. For example, temperature variations have been reported to alter the levels of gibberellin in different plant species [56, 57], which may affect the performance of *T*. *urticae* feeding on those plants [58]. Possibly, also the levels of other components are affected by DTR, which in turn could influence the performance of herbivores.

The resulting lower developmental thresholds (T_0_), as calculated by the linear model, suggest an effect of the amplitude of the temperature alternation, with a decreasing T_0_ as the amplitude of the temperature variation increased. The average temperature at which total immature development approximates zero is 3 to 4°C lower at DTR+15 compared to the constant temperature regime. This trend was confirmed by both nonlinear models. Degree-day modelling is a widely used tool to predict the timing of a range of biological processes and has been successfully adopted in the management of arthropod pests [28,59]. The amount of degree-days necessary to complete an event is calculated as the number of heat units above T_0_ [5]. Evidently, the number of degree-days required will increase as T_0_ decreases, as the organism will start to develop from a lower temperature onwards [7]. Our results highlight the impact of diurnal temperature variations, emphasizing the need to integrate temperature variations in predictive degree-day models. As such, population build-up of pests early in the season can be expected to happen earlier than when using constant temperature models. More in particular, in protected crops there is an increasing tendency to allow temperature variations within certain boundaries, as an energy-saving strategy [60, 61]. This so-called temperature integration approach allows greenhouse growers to save up to 20% of their energy costs [62,63]. Both the arthropod pests and their natural enemies introduced in the crop for their management are affected by these temperature variations, which may have its implications for the success of the biological control programmes. Our results demonstrate a substantial impact of temperature variations on the immature development of the studied mite species. For example, in a temperature range between 15°C and 25°C, the effect of a DTR+15 is more pronounced for *T*. *urticae*, resulting in a relatively faster development of the pest than of its predator *P*. *persimilis* as compared to a constant temperature regime (Fig 7). Differences were not only visible between pest and predator, but also among the studied phytoseiid predators (*P*. *persimilis* versus *N*. *californicus*). Between average daily temperatures of 15 and 25°C, *N*. *californicus* experienced a more positive effect on developmental rates at alternating temperatures than *P*. *persimilis*. Below an average daily temperature of 15°C, *P*. *persimilis* benefited more from alternating temperature regimes. Additionally, predation rates of the studied phytoseiid predators are also affected by temperature alternations (Vangansbeke et al., submitted). Therefore, we advocate that information on the impact of temperature variations should be included in models on biological control interactions as well as in the selection procedure of the most suitable natural enemies.

In summary, our results indicate that the rate summation effect alone does not suffice to explain the observed and predicted differences in developmental rates between constant and varying temperatures, especially in the lower and higher temperature ranges. Developmental rates and possibly other life history parameters at a specific temperature cannot be evaluated independently from the prevailing temperature regime. Further research should investigate possible physiological mechanisms that act in addition to the rate summation effect to fully appreciate the impact of diurnal cycling temperatures on life history traits of ectotherms. Diurnal temperature variations should be incorporated in predictive models on ectotherm ecology to generate more accurate predictions on the phenology of agricultural pests and their natural enemies. Finally, our findings may aid in further understanding the effects of climate change, as not only mean temperatures will increase, but also diurnal temperature ranges will be altered [64]. This is particularly relevant for ectotherms as they are more sensitive to temperature variation and are thus expected to be more vulnerable to the consequences of climate change [33, 65].

## Supporting Information

S1 Appendix Illustration of Jensen’s inequality for a hypothetical developmental rate-temperature curve of an ectothermic organism. (DOCX)

S2 Appendix Effect of different temperature regimes on the total developmental time of *Phytoseiulus persimilis*, *Neoseiulus californicus* and *Tetranychus urticae*. (DOCX)

S3 Appendix Estimated parameters of the Brière-2 and Lactin-2 model and corresponding evaluation criteria for total development of *Phytoseiulus persimilis*, *Neoseiulus californicus* and *Tetranychus urticae* at 4 diurnal temperature ranges. (DOCX)

S1 Data Data of female developmental times presented in separate working sheets. Working sheet (WS) 1: female developmental times of *P*. *persimilis*; WS 2: female developmental times of *N*. *californicus*; WS 3: female developmental times of *T*. *urticae*.
(XLSX)
