# Supplementary material for: Diurnal Temperature Variations Affect Development of a Herbivorous Arthropod Pest and its Predators
Source: PLoS One. 2015 Apr 15;10(4):e0124898. doi: 10.1371/journal.pone.0124898 (PMC4398551; doi:10.1371/journal.pone.0124898)
Supplement: S1 Appendix — (DOCX) [file pone.0124898.s001.docx]

Illustration of Jensen’s inequality for a hypothetical developmental rate-temperature curve of an ectothermic organism (Figure A.1.). At temperature x, the corresponding developmental rate derived from the nonlinear function equals f(x).

- Temperature range a_1_-a_2_, with an average temperature of a_1-2:_
  - Mean developmental rate a* > f(a_1-2_)
- Temperature range b_1_-b_2_, with an average temperature of b_1-2:_
  - Mean developmental rate b* = f(b_1-2_)
- Temperature range c_1_-c_2_, with an average temperature of c_1-2:_
  - Mean developmental rate c* < f(c_1-2_)


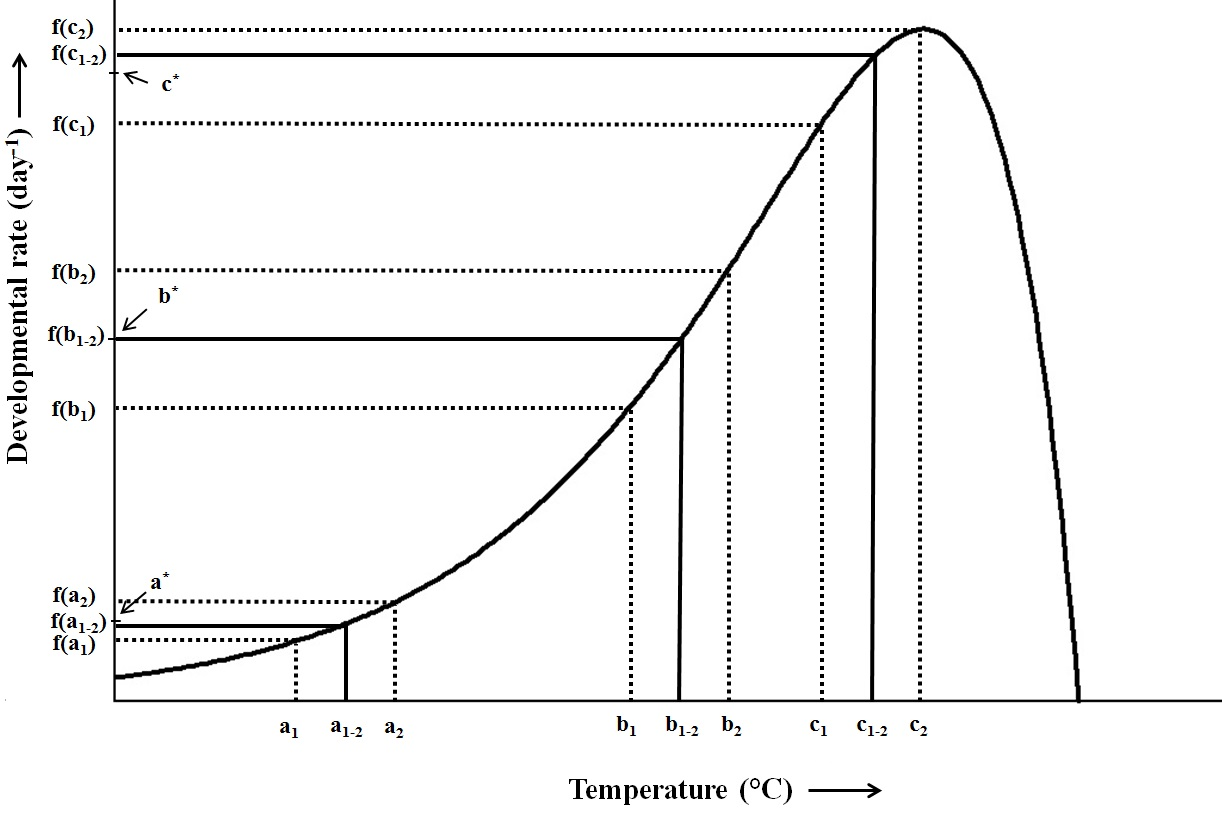


Figure A.1.: Developmental rate-temperature curve (after [Pradhan 1945](#_ENREF_56) and [Tanigoshi et al. 1976](#_ENREF_68)). a_1-2_ is the mean temperature of a_1_ and a_2_ with a corresponding developmental rate of f(a_1-2_). a* is the mean developmental rate [f(a_1_)+f(a_2_)]/2.
