# Supplementary material for: Diurnal Temperature Variations Affect Development of a Herbivorous Arthropod Pest and its Predators
Source: PLoS One. 2015 Apr 15;10(4):e0124898. doi: 10.1371/journal.pone.0124898 (PMC4398551; doi:10.1371/journal.pone.0124898)
Supplement: S2 Appendix — (DOCX) [file pone.0124898.s002.docx]

Table B.1.: Effect of different temperature regimes on the total developmental time (means ± SE) of *Phytoseiulus persimilis*, *Neoseiulus californicus* and *Tetranychus urticae*

|  | Temperature (°C) | | | DTR (°C)^a^ | Average daily temperatre (°C) | Developmental time (D) (days)^c^ | | | | | |  |  |  |  |  |  |  |
| --- | --- | --- | --- | --- | --- | --- | --- | --- | --- | --- | --- | --- | --- | --- | --- | --- | --- | --- |
|  | Day (°C) | | Night (°C) |  |  | n^b^ | *P. persimilis* | n^b^ | *N. californicus* | n^b^ | *T. urticae* |  |  |  |  |  |  |  |
| 1 | 12.5 | | 12.5 | 0 | 12.5 | 27 | 24.41 ± 0.43 p | 18 | 28.78 ± 0.82 y | 21 | 42.83 ± 0.83 x |  |  |  |  |  |  |  |
| 2 | 15 | | 5 | 10 | 11.7 | 29 | 17.62 ± 0.25 o | 28 | 21.82 ± 0.33 x | 26 | 38.58 ± 0.21 w |  |  |  |  |  |  |  |
| 3 | 15 | | 10 | 5 | 13.3 | 28 | 18.29 ± 0.26 o | 22 | 18.61 ± 0.24 w | 22 | 34.32 ± 0.40 v |  |  |  |  |  |  |  |
| 4 | 15 | | 15 | 0 | 15 | 22 | 14.43 ± 0.27 n | 19 | 15.11 ± 0.37 u | 40 | 27.45 ± 0.20 t |  |  |  |  |  |  |  |
| 5 | 17.5 | | 2.5 | 15 | 12.5 | 32 | 13.88 ± 0.11 m | 27 | 17.19 ± 0.29 v | 21 | 30.40 ± 0.32 u |  |  |  |  |  |  |  |
| 6 | 17.5 | | 17.5 | 0 | 17.5 | 29 | 8.97 ± 0.12 j | 28 | 11.11 ± 0.16 t | 17 | 21.50 ± 0.15 s |  |  |  |  |  |  |  |
| 7 | 20 | | 5 | 15 | 15 | 20 | 10.70 ± 0.15 l | 27 | 10.67 ± 0.12 s | 34 | 20.38 ± 0.16 r |  |  |  |  |  |  |  |
| 8 | 20 | | 10 | 10 | 16.7 | 26 | 9.38 ± 0,15 k | 19 | 10.08 ± 0.16 r | 23 | 20.07 ± 0.19 r |  |  |  |  |  |  |  |
| 9 | 20 | | 15 | 5 | 18.3 | 27 | 7.59 ± 0.10 i | 28 | 9.39 ± 0.14 q | 23 | 17.67 ± 0.12 q |  |  |  |  |  |  |  |
| 10 | 20 | | 20 | 0 | 20 | 25 | 6.40 ± 0.10 h | 23 | 7.15 ± 0.20 p | 37 | 14.42 ± 0.12 p |  |  |  |  |  |  |  |
| 11 | 22.5 | | 22.5 | 0 | 22.5 | 34 | 5.12 ± 0.04 g | 21 | 5.81 ± 0.11 n | 18 | 11.61 ± 0.16 n |  |  |  |  |  |  |  |
| 12 | 25 | | 10 | 15 | 20 | 22 | 6.41 ± 0.08 h | 22 | 6.73 ± 0.12 p | 34 | 12.50 ± 0.08 o |  |  |  |  |  |  |  |
| 13 | 25 | | 15 | 10 | 21.7 | 26 | 5.23 ± 0.08 g | 30 | 6.10 ± 0.09 o | 31 | 11.69 ± 0.07 n |  |  |  |  |  |  |  |
| 14 | 25 | | 20 | 5 | 23.3 | 29 | 5.05 ± 0.05 g | 26 | 5.48 ± 0.08 m | 30 | 9.87 ± 0.08 l |  |  |  |  |  |  |  |
| 15 | 25 | | 25 | 0 | 25 | 27 | 3.87 ± 0.05 d | 26 | 4.65 ± 0.07 ij | 25 | 9.28 ± 0.07 j |  |  |  |  |  |  |  |
| 16 | 27.5 | | 12.5 | 15 | 22.5 | 26 | 5.00 ± 0.06 g | 28 | 5.21 ± 0.05 l | 25 | 10.76 ± 0.08 m |  |  |  |  |  |  |  |
| 17 | 27.5 | | 17.5 | 10 | 24.2 | 30 | 4.28 ± 0.06 f | 18 | 5.00 ± 0.06 k | 20 | 9.55 ± 0.07 k |  |  |  |  |  |  |  |
| 18 | 27.5 | | 22.5 | 5 | 25.8 | 22 | 4.02 ± 0.05 e | 14 | 4.46 ± 0.06 i | 21 | 8.55 ± 0.04 h |  |  |  |  |  |  |  |
| 19 | 27.5 | | 27.5 | 0 | 27.5 | 30 | 3.52 ± 0.04 b | 15 | 4.17 ± 0.08 fg | 24 | 7.63 ± 0.06 f |  |  |  |  |  |  |  |
| 20 | 30 | | 15 | 15 | 25 | 29 | 4.21 ± 0.08 ef | 21 | 4.83 ± 0.09 jk | 27 | 8.91 ± 0.07 i |  |  |  |  |  |  |  |
| 21 | 30 | | 20 | 10 | 26.7 | 24 | 3.83 ± 0.06 cd | 24 | 4.17 ± 0.06 fg | 26 | 7.52 ± 0.02 f |  |  |  |  |  |  |  |
| 22 | 30 | | 25 | 5 | 28.3 | 28 | 3.30 ± 0.06 a | 28 | 3.82 ± 0.06 de | 21 | 7.10 ± 0.04 e |  |  |  |  |  |  |  |
| 23 | 30 | | 30 | 0 | 30 | 34 | 3.34 ± 0.05 a | 22 | 3.86 ± 0.05 e | 30 | 6.28 ± 0.06 b |  |  |  |  |  |  |  |
| 24 | 32.5 | | 17.5 | 15 | 27.5 | 24 | 3.81 ± 0.08 cd | 26 | 4.19 ± 0.07 fgh | 29 | 8.03 ± 0.07 g |  |  |  |  |  |  |  |
| 25 | 32.5 | | 22.5 | 10 | 29.2 | 22 | 3.66 ± 0.07 c | 22 | 3.73 ± 0.08 cd | 21 | 6.88 ± 0.07 d |  |  |  |  |  |  |  |
| 26 | 32.5 | | 27.5 | 5 | 30.8 | 29 | 3.38 ± 0.06 ab | 24 | 3.33 ± 0.07 a | 27 | 5.98 ± 0.02 a |  |  |  |  |  |  |  |
| 27 | 32.5 | | 32.5 | 0 | 32.5 | 26 | 4.25 ± 0.11 ef | 25 | 3.41 ± 0.06 ab | 26 | 6.20 ± 0.10 ab |  |  |  |  |  |  |  |
| 28 | 35 | | 20 | 15 | 30 | 15 | 6.50 ± 0.19 h | 22 | 4.25 ± 0.06 gh | 24 | 7.75 ± 0.13 fg |  |  |  |  |  |  |  |
| 29 | 35 | | 25 | 10 | 31.7 | 22 | 4.05 ± 0.09 de | 35 | 4.20 ± 0.06 fgh | 27 | 6.63 ± 0.11 cd |  |  |  |  |  |  |  |
| 30 | 35 | | 30 | 5 | 33.3 | 17 | 4.00 ± 0.09 de | 26 | 3.56 ± 0.05 bc | 24 | 6.04 ± 0.07 a |  |  |  |  |  |  |  |
| 31 | 35 | | 35 | 0 | 35 | / | / | 17 | 4.03 ± 0.10 ef | 9 | 7.50 ± 0.14 f |  |  |  |  |  |  |  |
| 32 | 37.5 | | 22.5 | 15 | 32.5 | / | / | 14 | 5.04 ± 0.11 kl | 23 | 7.54 ± 0.11 f |  |  |  |  |  |  |  |
| 33 | 37.5 | | 27.5 | 10 | 34.2 | / | / | 21 | 4.67 ± 0.13 i | 23 | 6.83 ± 0.13 d |  |  |  |  |  |  |  |
| 34 | 37.5 | | 32.5 | 5 | 35.8 | / | / | 6 | 4.75 ± 0.36 hi | 16 | 6.31 ± 0.17 abc |  |  |  |  |  |  |  |
| 35 | 37.5 | | 37.5 | 0 | 37.5 | / | / | / | / | / | / |  |  |  |  |  |  |  |
| 36 | 40 | | 25 | 15 | 35 |  |  | / | / | / | / |  |  |  |  |  |  |  |
| 37 | 40 | | 30 | 10 | 36.7 |  |  | / | / | / | / |  |  |  |  |  |  |  |
| 38 | 40 | | 35 | 5 | 38.3 |  |  | / | / | / | / |  |  |  |  |  |  |  |
| ^a^ DTR= diurnal temperature range (°C)  ^b^ n= number of females that successfully developed to adult  ^c^ Means (± SE) within a column followed by the same letter are not significantly different (P>0.05, Kruskal-Wallis, means  were separated using Mann-Whitney tests) | | | | | | | | | | |  |  |  |  |  |  |  |  |
