# Supplementary material for: Diurnal Temperature Variations Affect Development of a Herbivorous Arthropod Pest and its Predators
Source: PLoS One. 2015 Apr 15;10(4):e0124898. doi: 10.1371/journal.pone.0124898 (PMC4398551; doi:10.1371/journal.pone.0124898)
Supplement: S3 Appendix — (DOCX) [file pone.0124898.s003.docx]

Table 1: Estimated parameters (±SE) of the Brière-2 and Lactin-2 model and corresponding evaluation criteria for total development of *Phytoseiulus persimilis*, *Neoseiulus californicus* and *Tetranychus urticae* at 4 diurnal temperature ranges (DTR).

|  | *P. persimilis* | | | | *N. californicus* | | | | *T. urticae* | | | |
| --- | --- | --- | --- | --- | --- | --- | --- | --- | --- | --- | --- | --- |
|  | DTR0 | DTR+5 | DTR+10 | DTR+15 | DTR0 | DTR+5 | DTR+10 | DTR+15 | DTR0 | DTR+5 | DTR+10 | DTR+15 |
| **Brière-2** |  |  |  |  |  |  |  |  |  |  |  |  |
| a | 0.0003 ± 0.0000219 | 0.0003 ± 0.0000401 | 0.0003 ± 0.0000332 | 0.0003 ± 0.000017 | 0.0002 ± 0.000039 | 0.0002 ± 0.000022 | 0.0002 ± 0.00001108 | 0.0001 ± 0.000007 | 0.0002 ± 0.000008 | 0.0001 ± 0.000021 | 0.0001 ± 0.000021 | 0.00009 ± 0.000003 |
| T_0_ | 8.6323 ± 0.7035 | 6.8228 ± 2.4626 | 4.4634 ± 1.6586 | -0.7012 ± 1.4968 | 7.3540 ± 1.4526 | 7.3687 ± 1.7074 | 6.6235 ± 1.7799 | 5.6214 ± 1.2741 | 6.1661 ± 1.4257 | 6.2958 ± 2.2196 | 4.2534 ± 2.3079 | 0.3548 ± 1.6866 |
| T_L_ | 33.1147 ± 0.2432 | 33.9198 ± 0.5946 | 32.9537 ± 0.7228 | 30.0013 ± 0.0007 | 36.4915 ± 0.9132 | 36.4035 ± 0.3302 | 36.6661 ± 0.0017 | 35.8244 ± 0.0035 | 35.1955 ± 0.1790 | 37.1671 ± 1.1029 | 35.6531 ± 1.2038 | 35.8330 ± 0.1377 |
| d | 3.7773 ± 0.5929 | 4.4301 ± 1.8358 | 3.7546 ± 1.0791 | 10.6917 ± 0.7996 | 3.3894 ± 1.0194 | 3.5695 ± 0.7829 | 2.1471 ± 0.2321 | 1.8359 ± 0.1422 | 6.9259 ± 1.9145 | 4.5173 ± 1.8670 | 4.4539 ± 1.9186 | 3.3140 ± 0.3769 |
| R² | 0.9974 | 0.99 | 0.997 | 0.9976 | 0.9936 | 0.9928 | 0.9854 | 0.9924 | 0.9954 | 0.9941 | 0.9952 | 0.9977 |
| R² _adj_ | 0.9961 | 0.9826 | 0.9948 | 0.9959 | 0.9908 | 0.9885 | 0.9767 | 0.9878 | 0.9934 | 0.9906 | 0.9923 | 0.9963 |
| RSS | 0.0002 | 0.0006 | 0.0001 | 0.00008 | 0.0005 | 0.0004 | 0.0007 | 0.0003 | 0.0001 | 0.0001 | 0.000082249 | 0.000025064 |
| AIC | -100.1977828 | -67.98417956 | -82.31825531 | -84.10340372 | -101.9867751 | -82.19143529 | -77.1548932 | -84.78057395 | -119.6905921 | -94.66808454 | -96.42685513 | -107.1217232 |
| T_opt_ | 29.83 | 30.87 | 29.36 | 28.65 | 32.33 | 32.44 | 30.44 | 28.82 | 33.05 | 33.81 | 32.28 | 31.16 |
| D_r.opt_ | 0.303 | 0.307 | 0.278 | 0.273 | 0.285 | 0.295 | 0.260 | 0.239 | 0.166 | 0.170 | 0.156 | 0.133 |
| **Lactin-2** |  |  |  |  |  |  |  |  |  |  |  |  |
| ρ | 0.0139 ± 0.0005 | 0.0128 ± 0.0007 | 0.0116 ± 0.0005 | 0.0129 ± 0.0024 | 0.0110 ± 0.0004 | 0.0120 ± 0.0006 | 0.0108 ± 0.0008 | 0.0110 ± 0.0005 | 0.0068 ± 0.0003 | 0.0067 ± 0.0003 | 0.0062 ± 0.0003 | 0.0054 ± 0.0002 |
| T_L_ | 38.1573 ± 0.2250 | 38.6998 ± 0.3126 | 36.6978 ± 0.2271 | 36.5505 ± 1.0625 | 40.00 ± 0.2115 | 41.9620 ± 0.3421 | 40.1049 ± 0.4409 | 40.2740 ± 0.2769 | 40.9589 ± 0.3896 | 41.1241 ± 0.3136 | 39.3645 ± 0.3331 | 39.1898 ± 0.3004 |
| λ | -1.1575 ± 0.0123 | -1.1343 ± 0.0202 | -1.0936 ± 0.0130 | -1.1140 ± 0.0498 | -1.1104 ± 0.0102 | -1.1302 ± 0.0179 | -1.0919 ± 0.0196 | -1.0872 ± 0.0101 | -1.0741 ± 0.0084 | -1.0682 ± 0.0090 | -1.0534 ± 0.0080 | -1.0356 ± 0.0048 |
| ΔT | 2.4500 ± 0.1839 | 2.2095 ± 0.2518 | 1.8573 ± 0.1731 | 2.9801 ± 0.8846 | 1.8463 ± 0.1593 | 2.8176 ± 0.2796 | 2.5073 ± 0.3368 | 3.2379 ± 0.2262 | 1.9220 ± 0.2250 | 1.5743 ± 0.1827 | 1.4573 ± 0.1859 | 1.7333 ± 0.1629 |
| R² | 0.9962 | 0.9939 | 0.9963 | 0.962 | 0.9949 | 0.9936 | 0.9885 | 0.9974 | 0.9905 | 0.994 | 0.9935 | 0.9972 |
| R² _adj_ | 0.9943 | 0.9893 | 0.9935 | 0.9336 | 0.9927 | 0.9898 | 0.9817 | 0.9958 | 0.9864 | 0.9904 | 0.9897 | 0.9955 |
| RSS | 0.0004 | 0.0006 | 0.0003 | 0.0021 | 0.0005 | 0.0006 | 0.0008 | 0.0002 | 0.0003 | 0.0002 | 0.0002 | 0.000050435 |
| AIC | -93.26631104 | -67.98417956 | -73.529357 | -57.96207581 | -101.9867751 | -78.54224932 | -75.95311067 | -88.42975992 | -107.6058569 | -88.42975992 | -88.42975992 | -100.8284478 |
| T_0_ | 10.517 | 9.8417 | 7.7256 | 8.3798 | 9.5213 | 10.203 | 8.1509 | 7.6072 | 10.516 | 9.8483 | 8.3969 | 6.484 |
| T_opt_ | 29.5849 | 30.592444 | 29.41323 | 26.451389 | 32.65642352 | 32,08493723 | 30,80119532 | 29,07701184 | 32,5118906 | 33,88175206 | 32,443 | 31,01648 |
| D_r,opt_ | 0.300 | 0.303 | 0.283 | 0.239 | 0.293 | 0.290 | 0.265 | 0.239 | 0.157 | 0.173 | 0.158 | 0.136 |
